# Plant choice between arbuscular mycorrhizal fungal species results in increased plant P acquisition

**DOI:** 10.1371/journal.pone.0292811

**Published:** 2024-01-31

**Authors:** Sören Eliot Weber, Jordi Bascompte, Ansgar Kahmen, Pascal A. Niklaus

**Affiliations:** 1 Institute for Evolutionary Biology and Environmental Studies, University of Zürich, Zürich, Switzerland; 2 Departement Umweltwissenschaften, University of Basel, Basel, Switzerland; Universidade Federal de Minas Gerais, BRAZIL

## Abstract

Arbuscular mycorrhizal fungi (AMF) are plant root symbionts that provide phosphorus (P) to plants in exchange for photosynthetically fixed carbon (C). Previous research has shown that plants—given a choice among AMF species—may preferentially allocate C to AMF species that provide more P. However, these investigations rested on a limited set of plant and AMF species, and it therefore remains unclear how general this phenomenon is. Here, we combined 4 plant and 6 AMF species in 24 distinct plant-AMF species compositions in split-root microcosms, manipulating the species identity of AMF in either side of the root system. Using ^14^C and ^32^P/^33^P radioisotope tracers, we tracked the transfer of C and P between plants and AMF, respectively. We found that when plants had a choice of AMF species, AMF species which transferred more P acquired more C. Evidence for preferential C allocation to more beneficial AMF species within individual plant roots was equivocal. However, AMF species which transferred more P to plants did so at lower C-to-P ratios, highlighting the importance both of absolute and relative costs of P acquisition from AMF. When plants had a choice of AMF species, their shoots contained a larger total amount of P at higher concentrations. Our results thus highlight the benefits of plant C choice among AMF for plant P acquisition.

## Introduction

Arbuscular mycorrhizal fungi (AMF) are plant symbionts that colonize roots and provide plants with increased access to soil nutrients, particularly phosphorus (P) [1]. In exchange, plants provide carbon (C) to AMF in the form of sugars and fatty acids. The dependency of the symbiotic partners is asymmetric, however. As obligate biotrophs, AMF acquire C strictly from their host plants. Plants can acquire soil P through their roots, but P acquisition through AMF is generally more C efficient than direct root uptake, particularly at low soil P concentrations [2, 3]. AMF, on the other hand, consume 2–20% of plant photosynthates [1, 4]. Despite this high C investment by plants in AMF, plants generally benefit from this symbiosis [5], and accordingly plants acquire the majority of P through associated AMF [6–8].

Plant roots are typically colonized by multiple AMF species, not all of which are equally mutualistic. In fact, AMF species range from mutualistic to parasitic with respect to their effect on plant growth [9]. When a single AMF species is associated with a plant, the growth of plant and AMF are tightly linked: higher P supply by the AMF to the plant will increase the plant’s growth, and in turn the plant will be able to return more assimilates to the AMF, thereby increasing the AMF’s growth as well. However, when multiple AMF species interact with a host plant, this creates the possibility that an AMF species could ‘cheat’ by receiving C from the plant despite contribution comparatively little to the plant’s P supply. Such cheating could directly reduce the growth of the host plant, and indirectly also reduce the benefit the non-cheating AMF derive from the symbiosis. By acquiring C while investing relatively lower amounts of resources into the symbiosis, the cheating AMF species thus could gain a competitive advantage over the non-cheating AMF species. Cheating therefore should ultimately promote a breakdown of the plant-AMF mutualism unless mechanisms operate that allow plants to choose their AMF partners based on the resource benefit they provide. The persistence (>400 My), frequency (71% of modern vascular plants species) and generally mutualistic nature of the AMF symbiosis indicate that some form of partner choice indeed happens. Although the specific mechanisms remain unclear, plants are believed to either reward beneficial or sanction detrimental AMF.

One framework to analyze the exchange of resources between plants and AMF is biological market theory [10, 11]. As in a human market, plants are modeled as trading assimilated C for P provided by AMF and vice versa. If plants exert “optimal partner choice”, they are expected to preferentially allocate C to AMF which provide more P. Using petri dishes in which non-photosynthetic root organs of *Medicago truncatula* were colonized by different AMF species, Kiers et al. [11] found that plants allocate more C to AMF species that provide greater benefits in terms of P acquisition and resulting plant growth. This finding has since been corroborated in microcosm studies with photosynthesizing plants grown in soil with a choice of different AMF species [12, 13]. If AMF possess ‘behavioral plasticity’, they may respond to a reduced plant C supply by becoming more mutualistic, i.e. by returning more P to the plant per unit C obtained. In the market analogy, they would ‘lower the price’ of the commodity (P) they sell. The ratio of the amount of C a plant invests in an AMF network (hereafter referred to as ‘AMF individual’) to the units of P the AMF returns (the ‘price of P’ in units of C) is thus key to assessing the quality of AMF partners if providing P is the primary service AMFs provide to the plant. In addition to this C:P ratio, the absolute rate at which P is transferred to the plant is also likely to be important. A low C:P ratio alone would be of limited use to a plant if the rate of P delivery by the AMF cannot meet the plant’s needs, for example during periods of rapid growth. Thus, there must also be a match between the activities and growth rates of plants and their associated AMF.

This biological market regulation of the AMF symbiosis implies that as plants interact with a greater number of AMF species of varying quality, plants should allocate C to AMF species in a way that maximizes total P gain and P gain per unit C expended (or balances these goals if there is a trade-off between them). In turn, the lower price of C per unit P should allow plants to grow larger and thus have access to a greater amount of C to allocate to beneficial AMF species. Thus, increasing AMF species diversity (i.e. increasing AMF partner options for host plants) should result in a positive ‘biodiversity effect’ for both host plants and their associated AMF, as has been observed in some studies [14, 15].

As with all models, such market analogies are an abstraction (and simplification) of reality, and it is therefore important to evaluate whether these models capture the essence of the host-symbiont interaction. Indeed, it has been suggested that this may not be the case, since plant C may be a luxury good. It is also known that AMF provide benefits to plants other than P, including drought resistance and possibly protection against pathogens [16–18]. Nevertheless, the P-for-C trade is often assumed to be the core component of plant-AMF interactions; yet its dependence on plant choice have only been investigated in a relatively narrow set of plant-AMF species combinations. Thus, an important question is whether plant preference for AMF that yield more P per unit C supplied really is indeed a widespread and general phenomenon.

**Fig 1:**
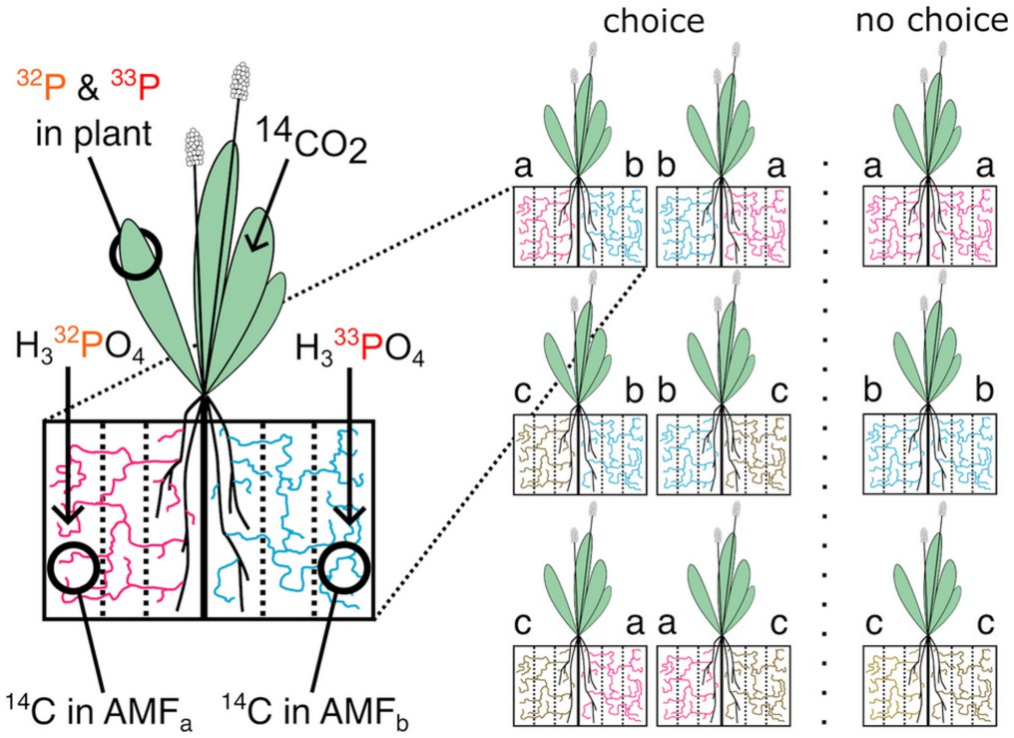
Experimental design. Plants and AMF were grown in microcosms with plant roots divided between two sides with a central plastic barrier. Mesh membranes (21μm pores) allowed AMF growth throughout each side of the microcosm. Plant roots were restricted to the innermost of the three compartments per side. The plant carbon (C) pool was labeled with ^14^C, and soil phosphorus (P) pools in the outermost compartments were labeled with either ^32^P or ^33^P. ^14^C transferred from plants to AMF was measured in the outer compartments of the microcosm. Radioactive P (*P) taken up by AMF from outer compartments and transferred to plants was measured in plant shoots. The space between inner and exterior compartments served as a buffer and prevented direct root uptake of applied *P from these outer compartments and direct root exudation of ^14^C into these outer compartments. Each plant species interacted with a pool of 3 AMF species: there were three choice microcosms with two AMF species and three no-choice microcosms with a single AMF species. Microcosms with 2 AMF species were replicated twice (with reciprocal ^32^P/^33^P labeling), resulting in 9 microcosms per host plant species.

Here, we used combinations of five host plant and six AMF species to test whether host plants preferentially allocate C to AMF that return higher amounts of P (Table 1). We used split-root microcosm to present two AMF species host plants in distinct parts of their root systems, and quantified C and P exchange using radioisotope tracers (Fig 1). We also investigated how plant nutrient budgets and growth depend on plant choice of AMF species. Specifically, we hypothesized that plants preferentially allocate C to AMF species that return more P, and that plants acquire more P and grow more when given a choice between AMF than when not.

**Table 1:** Plant and AMF species combinations used in this experiment with their respective taxonomic families.

Plant Species		AMF Monocultures	AMF Mixtures
Species pool 1			
*Holcus lanatus* (grass)	×	*Funneliformis mosseae* (Glomeraceae)	*F*. *mosseae × G*. *diaphanum*
*Lotus corniculatus* (legume)		*Glomus diaphanum* (Glomeraceae)	*F*. *mosseae × S*. *constrictum*
*Prunella vulgaris* (non-legume forb)		*Septoglomus constrictum* (Glomeraceae)	*G*. *diaphanum × S*. *constrictum*
Species pool 2			
*Lolium multiflorum* (grass)		*Rhizophagus invermaius* (Glomeraceae)	*R*. *invermaius* × *C*. *claroideum*
*Trifolium pratense* (legume)	×	*Claroideoglomus claroideum* (Claroideoglomeraceae)	*R*. *invermaius* × *D*. *celata*
*Plantago lanceolata* (non-legume forb)		*Diversispora celata* (Diversisporaceae)	*C*. *claroideum* × *D*. *celata*

Due to growth issues (see text) microcosms containing L. corniculatus and P. vulgaris were excluded from analysis.

## Materials and methods

### Experimental design

To generalize plant-AMF interactions across a functionally and phylogenetically broad set of species, we populated the split-root microcosms with different plant-AMF species compositions. For plant species, we chose 2 grasses (*Lolium multiflorum*, *Holcus lanatus*), 2 non-legume forbs (*Plantago lanceolata*, *Prunella vulgaris*), and 2 legumes (*Trifolium pratense*, *Lotus corniculatus*) commonly found in mesic managed European meadows. For AMF, we selected 6 species belonging to 4 families (Table 1). The plant and AMF species were each assigned to 2 pools with non-overlapping sets of species. Within each pool, all possible compositions of host plant and AMF species were realized (Table 1). The non-legume forb *P*. *vulgaris* did not germinate at a sufficient rate and the legume forb *L*. *corniculatus* failed to consistently grow roots on both sides of microcosms (described below), we thus ended up with 1 plant species in pool 1 instead of 3. In total, our experiment included 24 different compositions of host plant and AMF species (Table 1). The host plants were labelled with ^14^CO_2_, and the AMF associated with each half of the root system were labelled with ^32^P and ^33^P phosphate, respectively (Fig 1). The microcosms inoculated with 2 different AMF species were replicated twice, allowing the independent application of ^32^P and ^33^P to the other side of the microcosm (with the other AMF species). In total, the experiment consisted of 4 host plant species × (3 single-AMF species microcosms + 3 two-AMF species microcosms × 2 different P-labellings) = 36 microcosms.

### AMF inoculum

AMF inocula were prepared by inoculating propagating AMF cultures with the host plant *Plantago lanceolata* (cultures obtained from the Swiss AMF culture collection at Agroscope Reckenholz). To increase the host plant’s dependence on AMF and thus enhance AMF growth, we applied modified 10 mL Hoagland’s solution with ¼ strength P and additional N (0.125 mM KH_2_PO_4_, 1 mM NH_4_NO_3_) every 2 weeks [19]. After 7 months, we clipped plant shoots, air-dried the soils for 5 days, and mixed plant roots cut into small pieces with the dried soil. These dry inocula were stored at 4°C until use.

### Microcosms

Each microcosm had 6 compartments. A solid plastic divider was used to divide these microcosms into two sides, each with 3 compartments. The 3 compartments on each side were further subdivided by two 21-μm nylon mesh windows that allowed AMF hyphae, but not plant roots, to pass through (Fig 1). At the beginning of the experiment, the inner 4 of the 6 compartments were filled with a 9:1 mixture of quartz sand:meadow soil, both of which had been sterilized (the sand by autoclaving, the soil by irradiation with 75 kGy of high-energy X-rays). The 2 innermost compartments where roots would grow were inoculated with 15 mL of either the same or different AMF species inoculum (8% of soil in root compartments, inoculum described above). Then, 2 plant individuals were placed 4 cm apart on top of the central solid plastic divider so that half of their root system was directed to each side of the solid plastic divider. Ten mL of AMF-free soil microbial wash was applied to the root on each side of the microcosm to promote the establishment of a standardized soil bacterial community and to allow legume nodulation (indeed, *Trifolium pratense* and *Lotus corniculatus* were nodulated when the microcosms were harvested). This microbial wash was derived from an aqueous suspension of managed meadow soil, double-filtered through 11μm pore filter paper (MN 615, Macherey Nagel, Germany). The 2 outermost compartments were not filled until immediately before P isotope labeling (see below).

Plants were grown under P limitation while other macro- and micronutrients were provided by biweekly applications of the modified Hoagland’s solution described above. To prevent powdery mildew infection, which occurred in our greenhouse, 0.5% micronized sulfur was preemptively sprayed on plants every 2 weeks.

### Isotope labeling and harvest

Seventy days after starting the microcosms, we labeled soils with H_3_^32^PO_4_ and H_3_^33^PO_4_ and 14 days later plants with ^14^CO_2_ (S1 Fig). The chamber we used to apply the ^14^CO_2_ label was too small to accommodate all the microcosms at once. Therefore, we divided our microcosms into 5 blocks, each block corresponding to a different host plant species. These 5 blocks were labelled sequentially, with a 2–3 day interval between blocks.

To apply the P label, we first filled the two outermost compartments with the same 9:1 sand:soil mixture we used for the other compartments. We then injected approx. 800 kBq of either ^32^P or ^33^P phosphoric acid into these compartments using a shielded syringe.

Fourteen days later, we applied the ^14^C label by placing the microcosms in a climate-controlled, gas-tight chamber where the CO_2_ concentration was monitored with an infra-red gas analyzer (LI-840A, Li-Cor, NE). We placed an aqueous solution of Na_2_^14^CO_3_ in a glass bulb through which chamber air was circulated with a small pump. We released ^14^CO_2_ from this carbonate solution by repeatedly injecting sulfuric acid with a syringe, maintaining the chamber CO_2_ concentrations at about 400 ppm. When all ^14^CO_2_ was released, we continued to add ^12^CO_2_ instead. This procedure was followed for 2 photoperiods (2 days), during which plants were able to assimilate the ^14^CO_2_ released from the carbonate solution and, as the label started to cycle in the model systems, also from plant and soil microbial respiration. The chamber was then opened and ventilated, and the microcosms were kept in the greenhouse for another 5 days before destructive harvesting. First, the shoots were clipped. Then, all 6 below-ground compartments were collected separately, and roots were separated from soils and washed. Plant and soil biomass were oven-dried at 80 and 105°C, respectively.

### Quantification of radiolabels

Plant material was ashed in ceramic crucibles in a muffle oven (temperature ramped up over 6h to 600°C, maintained at this temperature for 3h, and then slowly cooled back to room temperature). In this process, C in the biomass was released as CO_2_, while P remained in the mineral ash to which 2 mL 0.1 M H_2_SO_4_ were added, followed by 5 mL H_2_O. This extract was filtered (MN 615, Macherey Nagel, Germany) and ^33^P and ^32^P activity in the extract was quantified by liquid scintillation counting (LSC; 1 mL aliquot in 4 mL Ultima Gold cocktail, Tricarb 2900 TR liquid scintillation counter, PerkinElmer, MA). β-decays were recorded separately in the 4–248 and 248–1710 keV energy window. ^33^P decays with a maximum energy of 248 keV and is therefore recorded exclusively in the low-energy windows. ^32^P decays with a maximum energy of 1709 keV, with 75.6% of these decays recorded in the high energy window and 24.4% in the low energy window. This known constant partitioning of ^32^P decays between the two energy windows allowed the determination of ^32^P and ^33^P activities from the two count rates. We later discovered that some ^14^C had remained in some samples despite ashing and acidification. We therefore re-counted all samples after the P radioisotopes had decayed to negligible levels (>21 half-lives) and adjusted all ^33^P estimates by subtracting this background.

We quantified the net amount of C transferred from the plants to the AMF as ^14^C activity in the labelling compartment. This measure included the label in live hyphae plus residues from the turnover of biomass such as cell walls. In brief, dry soil subsamples of approx. 1 g were taken from homogenized labeling compartment soil and combusted in an automated oxidizer (Model 307, Perkin Elmer, MA) in which the CO_2_ produced was trapped (CarboSorb, Perkin Elmer, MA). ^14^C activities were then determined by LSC (energy window 4–160 keV; PermaFluor LSC cocktail, Perkin Elmer). Although the combustion process separates C from P, we allowed the radioactive P isotopes to decay for 8 months prior to these measurements (approx. 8 and 16 half-lives for ^33^P and ^32^P, respectively).

### Quantification of shoot N and P

We measured shoot P concentrations colorimetrically (San++ continuous flow analyzer, Skalar, The Netherlands) in the same extracts used for radiophosphorus determination. We also determined shoot N concentrations in ground shoot material using an automated elemental analyzer (Flash EA, Thermo-Finnigan, Germany).

### Root colonization by AMF

Root samples were cleared in 5% KOH and AMF structures stained with India ink (5% solution in vinegar) following the Vierheilig and colleagues (1998) method prior to mounting root segments on microscope slides [20]. As this method of staining is as effective as traditional histological stains [20], we used this approach to avoid exposure to possible carcinogens. Then, we determined the percentage of root length colonized by AMF, using a crosshair reticle mounted in the eyepiece of a compound microscope at 200x magnification [21]. Specifically, we counted the number of root segments colonized by AMF within 50 randomly selected intersects.

### Data analysis

All root systems were adequately colonized by AMF. However, roots of *Lotus corniculatus* did not grow on both sides of the microcosm for two single-species AMF compositions. Because this violated our experimental assumptions and resulted in a lack of single-AMF reference microcosms for 2 of the 3 two-species AMF compositions for *L*. *corniculatus*, we excluded the microcosms containing *L*. *corniculatus* from the analyses. In addition, plants grown with the AMF species *Claroideoglomus claroideum* consistently had higher shoot P (both in this study and in a separate experiment using the same species pools), but this was not reflected in the radio-P transferred to plants by this AMF species. Because one of our assumptions was that transferred radio-P (i.e. short-term P transfer to plants) reflects average lifetime P transfer to plants (and thus the total shoot P pool), we present results including and excluding species compositions that contain *C*. *claroideum*.

Our goal was to identify general patterns that hold across different plant and AMF species. We therefore averaged data at the fundamental level of replication for these questions, i.e. the different plant species × AMF species combinations. After accounting for radioactive decay, the activities of ^32^P and ^33^P in plant shoots were similar (8.0 ± 2.1 kBq ^32^P; 7.1 ± 1.6 kBq ^33^P). Since we were not interested in the identity of these P isotopes other than to determine the P transfer from the different AMF species in each microcosm, we express the transferred radio-P label as *P.

In line with the biological market perspective adopted here, we were interested in the amount of C a plant transferred to AMF relative to the amount of P returned by the AMF. Operationally, we estimated this as the ratio of ^14^C recovered in a hyphal compartment to *P in the plant shoot transferred to the plant by the AMF growing in that compartment (^14^C:*P). This index essentially quantifies the plant C transferred to a given AMF to obtain a unit of P in return.

To rank AMF in terms of their benefit (quality) to the plant, we estimated the average effect of each AMF species on plant P supply using mechanistic diallel analysis. Diallel analysis estimates the average effect of AMF species across single and two AMF species compositions, which is statistically more robust than using only single AMF microcosms, which are only available at much lower replication. This average effect is known as the general combining ability (GCA). See S1 Appendix for further details.

At the microcosm level, we examined how shoot carbon and nutrient contents and radioisotope data differed between two-species AMF compositions and their respective single species compositions.

Within microcosm, we tested whether the exchange of radioisotope between sides depended on the relative quality of the two AMF present in microcosms. Specifically, we tested whether “trade” with the AMF increased when it had a higher quality rank.

All analyses were performed by fitting linear (mixed) models summarized in ANOVA tables. These models used plant species × AMF species composition as the level of replication.

## Results

### Relative quality of AMF

**Fig 2:**
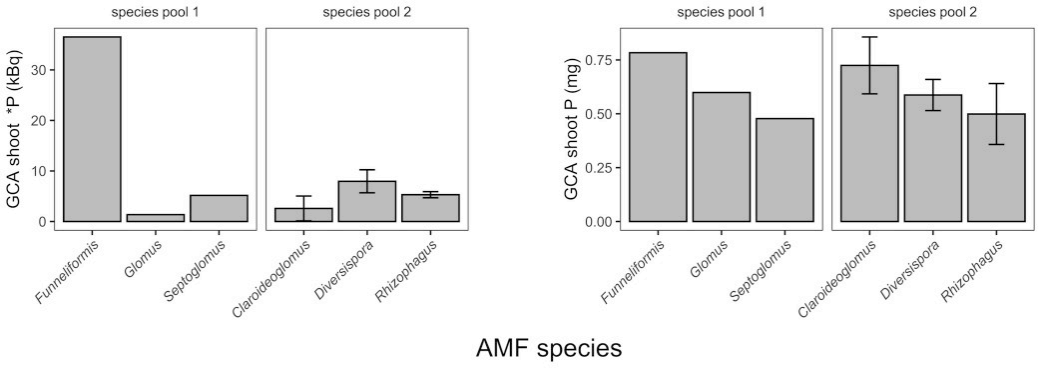
AMF quality indicators. We used general combining abilities (GCAs) for (A) shoot radioactive P (*P), and (B) shoot P as indicators of AMF quality (GCAs were estimated using mechanical diallel analyses; S1 Appendix and Methods). Error bars are ± 1 s.e. of the mean. AMF species are indicated by their genus; see Table 1 for full names.

The average effect of each AMF species (the so-called general combining ability, or GCA, Fig 2, S1 Appendix) was determined for shoot contents of *P (kBq) and total P (mg) (Fig 2). We interpreted the GCA of shoot P as the accumulated lifetime benefit of a given AMF species, whereas the GCA of shoot *P was interpreted as the benefit of *P transferred to the shoots during the comparably short radiolabeling period. The qualitative ranking of AMF species in GCA shoot P (mg) was *F*. *mosseae* > *G*. *diaphanum* > *S*. *constrictum* in species pool 1, and *C*. *claroideum* > *D*. *celata* > *R*. *invermaius* in species pool 2. The qualitative ranking of AMF species in GCA shoot *P (kBq) in species pool 1 was *F*. *mosseae* ≫ *G*. *diaphanum* ≅ *S*. *constrictum* and *D*. *celata* > *R*. *invermaius* > *C*. *claroideum* in species pool 2. The ranking based on the two GCA metrics were thus qualitatively broadly identical except for *C*. *claroideum* which was beneficial in the long-term, but this was not reflected in the *P label data.

### Radioisotope exchange

To test how host plants exchanged radioisotopes with specific AMF species, we used the plant-AMF-species composition, further divided by AMF identity, as replicate. In other words, in case of the two-AMF species microcosms, we considered the microcosm halves as replicates; in the case of the single-AMF microcosms, which had the same AMF species on both sides, we used the average of the two halves as replicate. AMF quality was described using the general combining abilities (GCA) for *P and P, as described above.

**Fig 3:**
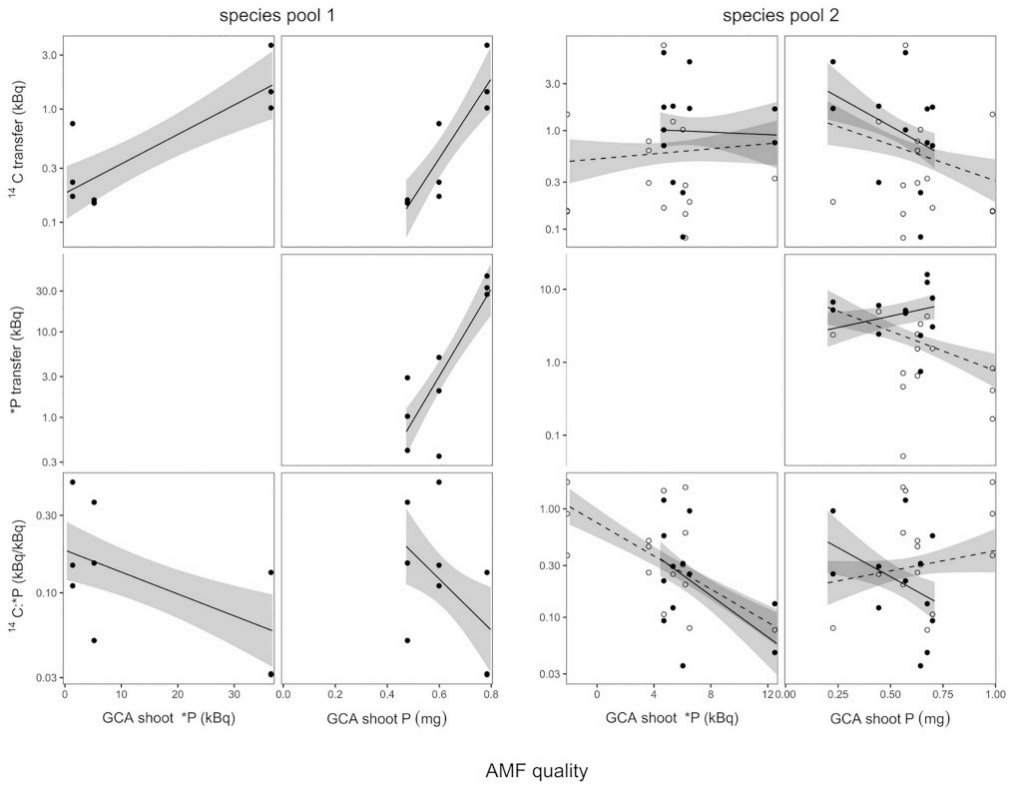
Dependence of radioisotope data on the relative quality of AMF. AMF quality was determined as general combining ability (GCA) using diallel analysis, for shoot radio-P (*P) and total shoot P (P). Radioisotope data refer to microcosm halves, i.e. they characterize the C and P exchange that occurs with each AMF species in each plant × AMF species composition. Solid regression lines and closed circles represent compositions without Claroideoglomus claroideum, dashed regression lines and open circles represent compositions with C. claroideum. See Methods for details. Gray area indicates ±1 s.e.

^14^C transfer from the plant host to AMF increased significantly with AMF quality determined as shoot *P GCA (Fig 3, F_1,26.6_ = 5.99, P = 0.021), i.e. plants transferred more recent assimilates to AMF which generally provided more *P to the plants. ^14^C:*P, i.e. the cost of P in units of C, also decreased with AMF quality (Fig 3, F_1,23_ = 9.2, P = 0.006). This decrease of ^14^C:*P with AMF quality (measured by *P GCA) was stronger in species pool 2 than in species pool 1 (Fig 3, F_1,23.3_, = 8.8, P = 0.007).

Qualitatively similar relationships for ^14^C were found when the GCA for total shoot P was used as AMF quality indicator in species pool 1 (^14^C: F_1,32_ = 9.23, P = 0.005 for pool × GCA P). As discussed above, species pool 2 contained the AMF *C*. *claroideum*, which was beneficial in the long-term, but this effect was not reflected in the short-term P flux assessment using *P. When this AMF was excluded from the analysis, a comparable marginally significant effect of shoot P GCA on ^14^C:*P (F_1,15.4_ = 3.9, P = 0.07) was found in species pool 2.

### Preferential radioisotope exchange with higher-quality AMF species

**Fig 4:**
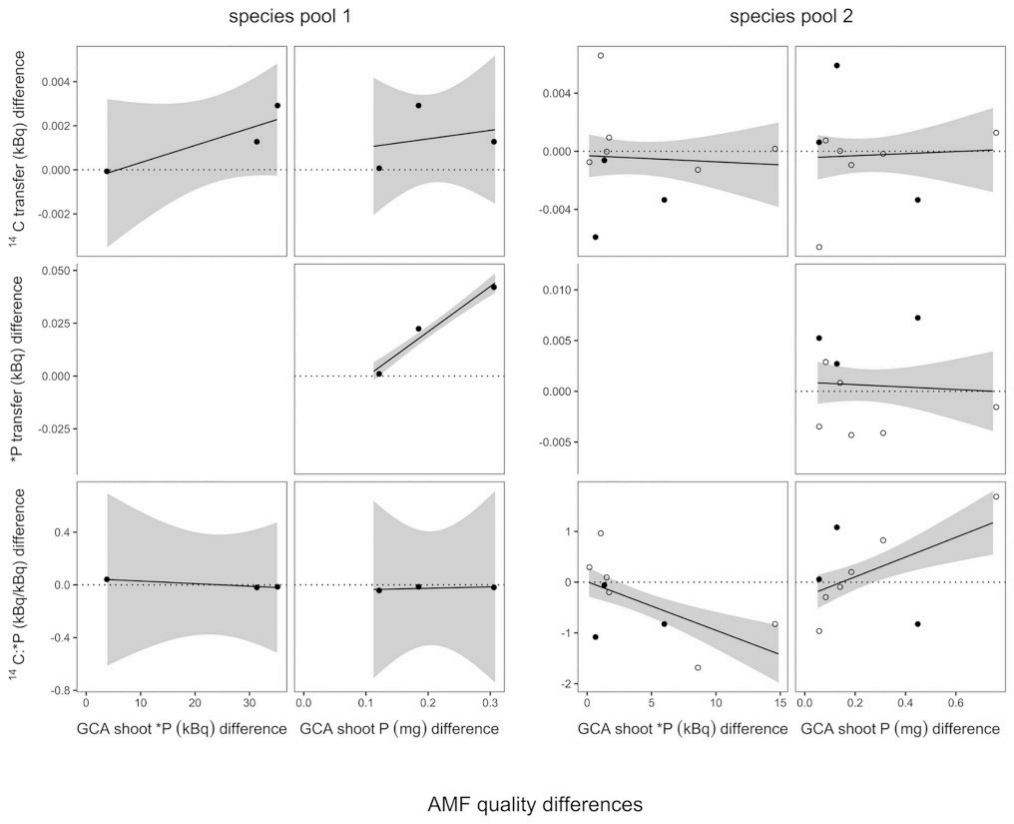
Difference in isotope exchange between microcosm sides, in dependence of relative AMF qualities. Relative AMF quality is either quantified as the GCA difference for shoot *P or total shoot P (see Methods for details). Relative ^14^C transfer is quantified as the difference in ^14^C allocation between microcosm sides with different AMF species. A positive slope indicates that C allocation is biased towards the side with the higher-quality AMF. ^14^C:*P quantifies the P costs in units of C. A negative slope indicates that these are lower on the side with the higher-quality AMF. Solid lines and gray areas indicate linear regression lines and standard errors. Open symbols indicate compositions containing the AMF Claroideoglomus claroideum for which short-term and long-term quality (assessed as *P transfer to shoots and total shoot P accumulation, respectively) differed substantially (see Methods for details).

To test whether plants allocated more ^14^C to relatively more beneficial AMF when given a choice, we regressed the difference in ^14^C transfer to both microcosm halves against the difference in AMF quality, using plant × AMF composition as the unit of replication (Fig 4).

Using GCA for shoot *P as an AMF quality indicator, in species pool 1^14^C transfer towards the side with the more beneficial AMF averaged higher, but this was statistically insignificant. Side differences in ^14^C:*P ratios remained unaffected.

Using GCA for shoot P as a quality indicator, we observed an increased transfer of *P towards the more beneficial microcosm side, but only in pool 1 containing a single plant species (AMF quality × species pool, F_1,8_ = 35.6, P < 0.001, Fig 4). Side differences in ^14^C:*P remained unaffected. In general, differences in plant C allocation to AMF in differing portions of the root system were limited.

### Net benefit of choice between AMF species

**Fig 5:**
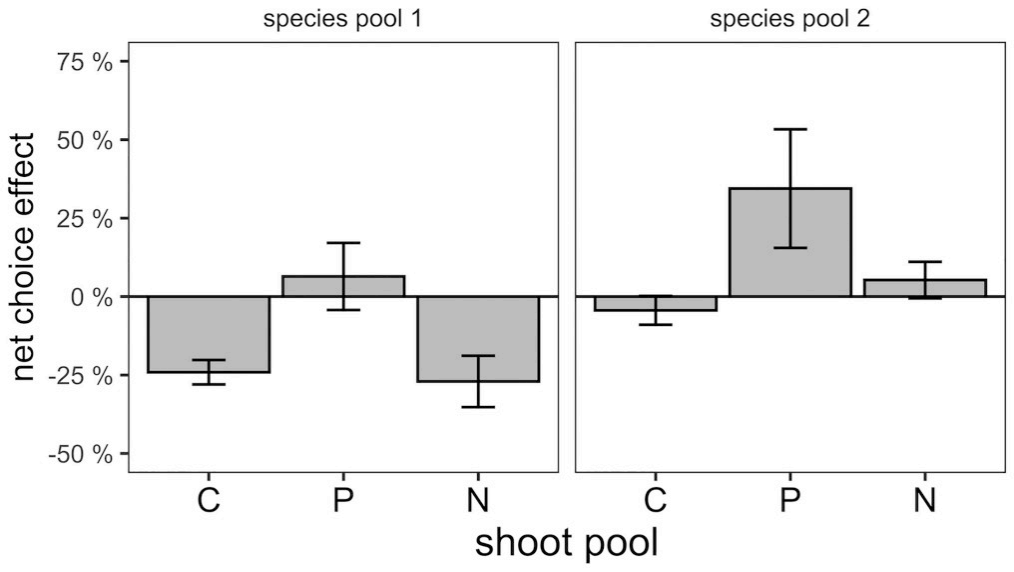
Effects of choice between AMF. Difference in shoot total carbon content (C), phosphorus (P) and nitrogen (N) content between choice and no-choice microcosms. Bars and error bars show means and standard errors, using species composition as unit of replication.

To assess the benefit a plant derives from interacting with two rather than just one AMF, we determined the difference in total shoot nutrients between two-AMF and single-AMF microcosms (Fig 5). Plants had a marginally significantly higher shoot P content when offered a choice between AMF species (t_11_ = 1.88, P = 0.087, Fig 5). Shoot N and C pools were on average lower in the choice treatments. As a result, shoot P concentration (g P / g C) was 41±14% higher in the two-AMF compositions (t_11_ = 2.96, P = 0.013) and shoot N:P (g N / g P) was marginally significantly reduced by 24 ± 12% (t_11_ = -1.99, P = 0.07).

**Fig 6:**
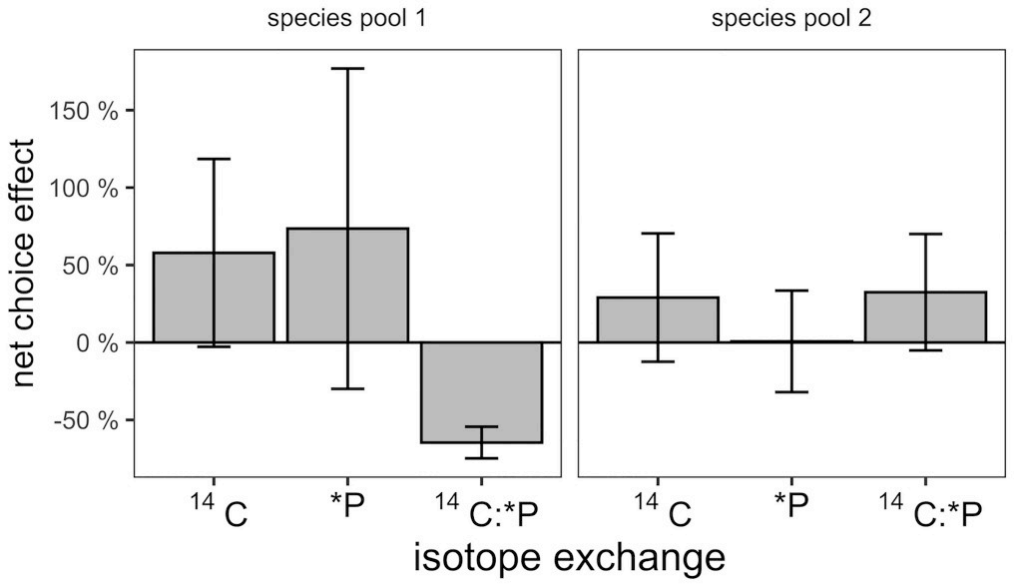
Percent differences in ^14^C transferred to AMF, radio-active P (*P) transferred from AMF to plants and ^14^C:*P between plants and AMF when plants did and did not have a choice between AMF species. Positive values reflect higher amounts in plants with a choice of AMF species, negative values the reverse. Error bars are ± 1 s.e. of the mean.

We assessed whether plant choice between AMF species altered patterns of plant ^14^C allocation to AMF, plant *P acquisition through AMF, and their ratio (Fig 6). ^14^C in AMF and *P returned to plant shoots averaged higher in the choice treatment, but this difference was not statistically significant. When examined separately in the two species pools, plants with a choice of AMF species had 65 ± 10% lower ^14^C:*P in species pool 1 (t_2_ = -6.34, P = 0.024, Fig 6).

## Discussion

We used a broad range of plant and AMF species to investigate the exchange of C and P in the AMF symbiosis and its consequences for plant P acquisition. This was achieved using split-root microcosms in which plants were labelled with ^14^C and AMF were supplied with ^32^P and ^33^P phosphate (*P). The AMF species we used differed in their effects on plant P nutrition. We found evidence that more beneficial AMF species acquired more C from plants, but we did not observe that individual plants preferentially allocated larger amounts of C to more beneficial AMF species when given a choice. However, we found that a choice between two AMF species resulted in an improved P supply, as evidenced by higher shoot P concentrations and P:N ratios.

Biological market theory predicts that the choice between partners of different quality should allow a host to optimize the exchange of resources with associated symbionts [10]. For biological markets to generate evolutionarily stable strategies, partners must be able to (1) discriminate partner quality and (2) preferentially allocate resources to better partners. Thus, plants are expected to detect quality differences between AMF species in their root systems and allocate more C to higher-quality AMF species [22, 23]. Through this mechanism, market-like competition between AMF species for preferentially allocated C is the way plants exert leverage over AMF to acquire more P at lower rates of C investment. Although studied at the level of AMF species, it is likely that preferential allocation extends to the genotype and perhaps even the individual level.

An important question is under what circumstances host plants can discriminate between AMF species and preferentially allocate C to the more beneficial symbiont. Published experimental data suggest that this is not always the case and that this ability may depend on a spatial segregation of AMF (e.g. in different segments of a larger root system). Bever et al. [24] showed that the coarse-rooted plant species *Allium vineale* could preferentially allocate C to higher quality AMF species, but this plant was not able to preferentially allocate C when AMF species were not spatially separated by a barrier. Kiers et al. [11] used non-photosynthetic root organs of the legume *Medicago truncatula* interacting with different AMF species and found that these root organs transferred more C to AMF species that provided more P. The ability of *Medicago truncatula* root organs to preferentially allocate C remained even when AMF species were not spatially separated [11]. Argüello et al. [13] found that neither the legume *Trifolium pratense* nor the forb *Plantago lanceolata* showed large differences in C allocation to AMF species of different quality. However, Argüello et al. [13] found that the lower quality AMF species increased P transfer to both plant species when a higher quality AMF species was present on the opposite side of the root system. During short time periods of fluctuating P availability, van’t Padje, Werner & Kiers [25] found that AMF can control the rate of C for P exchange by storing “excess” P, thereby maintaining plant P demand.

The effect of including multiple AMF species in a system, and thus allowing plants to choose among symbionts, can be considered an “AMF biodiversity effect”. Biodiversity effects typically result from the combination of functionally distinct organisms in a community. In our study, we used a range of AMF taxa from functionally distinct families that differ in life history traits that underlie many aspects of their mutualistic quality to plants [26, 27]. Variation among AMF species in growth rates and patterns of biomass allocation results in differences among taxa in the timing and amount of P they provide to plants. AMF species also differ in the maximum distance from plant roots at which they can acquire soil P [28]. Variation in AMF traits, such as relative biomass allocation to within or outside of roots and growth rates influence the degree to which plants benefit from AMF assemblages [26, 29]. Functional trait differences among AMF species may determine their relative quality to the host; however, these differences could also lead to functional complementarity among AMF species, over time or space, which could also contribute to increased overall P supply to host plants. In other words, it may be beneficial for a host plant not only to invest C in the single most beneficial AMF species, but also to support a greater diversity of AMF, even if these are less beneficial when considered in isolation. Finally, in addition to differences in the spatio-temporal characteristics of P uptake from soils and the exchange of this P for plant C [28, 30], AMF species also differ in other functions they provide to plants [15, 31]. Thus, the benefits of AMF diversity to plants may well be rooted in multifunctional complementarity that cannot be fully understood by focusing on P dynamics alone.

In our study, the transfer of applied P radioisotope tracer (*P) broadly paralleled the effects observed on shoot P, i.e. AMF species that transferred greater amounts of *P to plants between label application and shoot harvest also caused greater increases in total shoot P. We interpret this as the lifetime benefits of an AMF paralleling the short-term benefits assessed by the radiolabel. An exception to this rule was the AMF *Claroideoglomus claroideum*. This species had greater positive effects on plant P than the other AMF to which it was compared, an effect we confirmed in an independent greenhouse experiment. However, this benefit did not extend to the observed transfer in *P. Several mechanisms could explain this discrepancy: first, AMF species may differ in growth rate and temporal allocation patterns of C; if this were true, then *C*. *claroideum* would have had greater beneficial effects at the beginning of the experiment, prior to label application. A second possibility is more technical than biological. To ensure that shoot *P was derived from AMF and not from direct root uptake, *P was applied a few centimeters away from the compartments containing plant roots. If *C*. *claroideum* acquired a larger fraction of P in the vicinity of plant roots than the other AMF, then the benefits of *C*. *claroideum* would indeed have manifested themselves more strongly in total P than in *P. This possibility is supported by evidence that *C*. *claroideum* hyphal density and *P uptake decrease more rapidly with distance from plant roots than in other AMF species [32]. Finally, the radiolabel used may have differed in other characteristics (e.g. chemical form, soil depth, concentrations) from native soil P, which could explain the different ratio of observed P and *P transfer in *C*. *claroideum* compared to the other AMF species.

In our study, we attempted to limit plant growth by P availability, primarily by applying P-deficient Hoagland’s solution with N:P of 61.5 (g/g). Increased tissue P concentrations when a choice of AMF species was available indicated that a diversity of available AMF did improve plant P nutrition. However, shoot biomass did not increase, possibly because its variability was simply so great that statistical power was lost. In any case, plants grown with a choice of AMF had shoot N:P ratios of 10.9 ± 0.5 g N (g P)^-1^, which is well within the range in which N, P, or both nutrients usually limit productivity of dry grasslands ([33] cited in [34]).

Here, we tested six AMF species from three taxonomic families known to be functionally distinct, extending previous studies of preferential allocation that focused on smaller sets of species [11–13, 24]. However, our study focused heavily on AMF species from Glomeraceae, a family known for rapid growth and high allocation to intra-radical structures [29]. AMF from different families exhibit contrasting growth responses to different host plant species and in patterns of biomass allocation (root colonization vs. soil exploration vs. spore production). In turn, plant species benefit differently (in terms of growth) from associating with AMF in general or with different AMF species. Future studies on this topic should take a more comprehensive and comparative approach, testing preferential allocation between plants and fungal species from more families than we did here. Such an approach would develop upon the general patterns we have described and exceptions to be understood in more detail than is possible with the current number of plant and AMF families evaluated to date.

## Supporting information

S1 Appendix Diallel analysis/general combining ability estimation. (PDF)

S1 Fig Experimental timeline. (TIF)
