# Supplementary material for: Plant choice between arbuscular mycorrhizal fungal species results in increased plant P acquisition
Source: PLoS One. 2024 Jan 31;19(1):e0292811. doi: 10.1371/journal.pone.0292811 (PMC10830030; doi:10.1371/journal.pone.0292811)
Supplement: S1 Appendix — (PDF) [file pone.0292811.s001.pdf]

S1 Appendix.

AMF quality estimation

Background

Plant preferences for AMF species are expected to depend on their relative quality. To investigate plant choice in the present experiment, we therefore derived AMF quality metrics. To do so, we used mechanistic diallel analysis, an approach originally developed for plant breeding (Griffing 1956). The diallel model predicts the performance of a mixed system as sum of the general combining abilities of the parts (the GCAs), plus specific combining abilities (SCAs), terms modeling the non-additive (interaction) effects that occur in the mixture. This approach has been adapted by biodiversity researchers (e.g. Wuest and Niklaus 2018) as an alternative to using species monocultures as 'reference' values when replication of any one composition is low, as in the design of the present experiment. For example, there were 9 microcosms per species pool and host plants (Fig. 1 Main Text). Designating the AMF species as A, B and C, based on single-AMF microcosms only, the quality rank of AMF A would depend the microcosm with AMF composition A-A only. In contrast, the GCA of AMF A depends on 5 microcosms (the ones with AMF compositions A-A, A-B, A-C, B-A, C-A; c.f. Fig 1. Main Text) and hence is statistically more robust.

We calculated GCAs for plant shoot phosphorus, since our premise is that P supply is the primary benefit of a plant associated with AMF. We determined GCAs separately for total shoot P (mg), and for shoot radio-P (*P in the Main Text; kBq). The former reflects the lifetime benefit of associating with AMF, the latter the benefit derived during the radiolabelling period.

Estimation

We determined GCAs as model coefficients in a linear model with a single 3-degree-of-freedom term and no global intercept. The contrasts in this term were encoded as shown in Table 1. Note that for the single-AMF microcosms, the value of the respective GCA is taken twice because the AMF occurs in both microcosm sides.

References

Griffing, B. 1956. “Concept of General and Specific Combining Ability in Relation to Diallel Crossing Systems.” Australian Journal of Biological Sciences. https://doi.org/10.1071/bi9560463.

Wuest, Samuel E., and Pascal A. Niklaus. 2018. “A Plant Biodiversity Effect Resolved to a Single Chromosomal Region.” *Nature Ecology & Evolution* 2 (12): 1933–39.

Table 1. Contrast encoding in GCA term.

Microcosm	AMF composition	a	b	c
1	a_a	2	0	0
2	b_b	0	2	0
3	c_c	0	0	2
4	a_b	1	1	0
5	a_c	1	0	1
6	b_c	0	1	1
7	a_b	1	1	0
8	a_c	1	0	1
9	b_c	0	1	1
