# Supplementary figures and images for: Plant choice between arbuscular mycorrhizal fungal species results in increased plant P acquisition
Source: PLoS One. 2024 Jan 31;19(1):e0292811. doi: 10.1371/journal.pone.0292811 (PMC10830030; doi:10.1371/journal.pone.0292811)

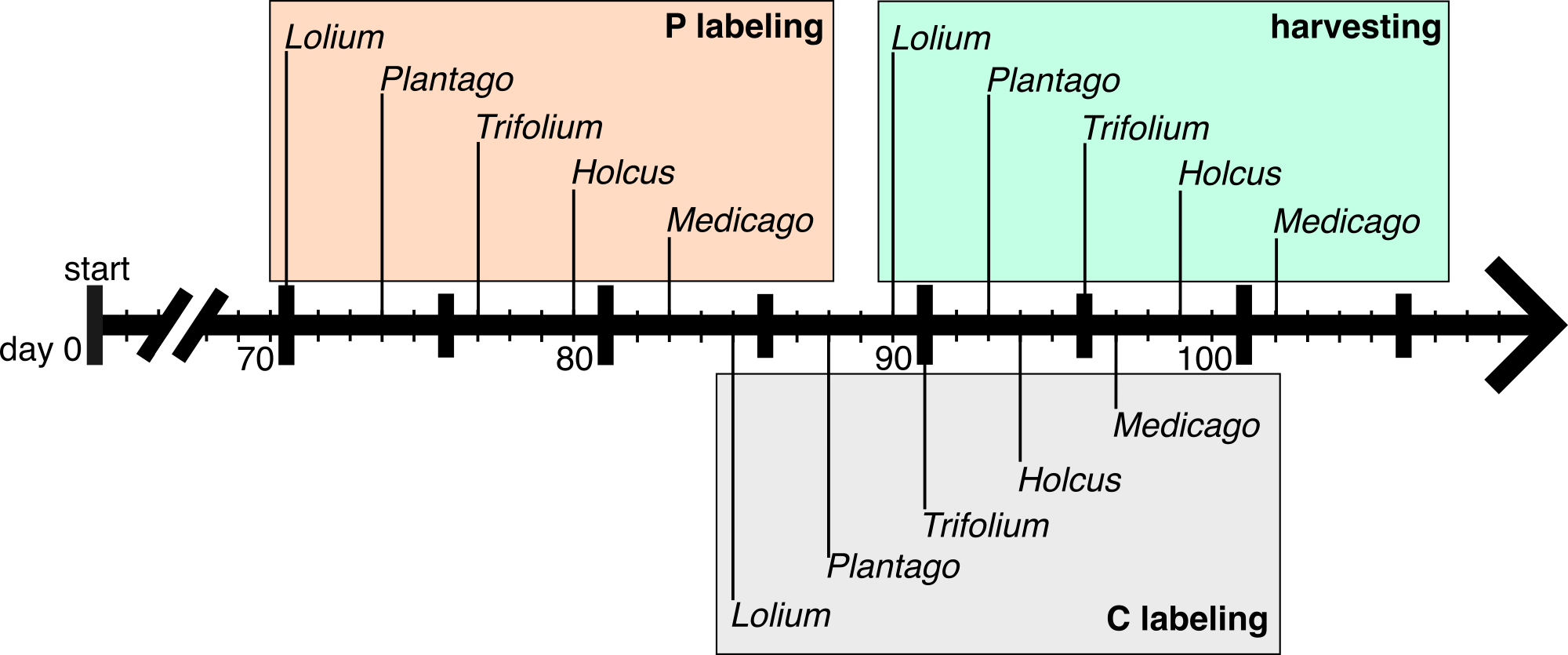

Supplement: S1 Fig — (TIF) [file pone.0292811.s002.tif]
